# Pneumonic Plague, Northern India, 2002 

**DOI:** 10.3201/eid1304.051105

**Published:** 2007-04

**Authors:** Manohar Lal Gupta, Anuradha Sharma

**Affiliations:** * Indira Gandhi Medical College, Shimla, Himachal Pradesh, India

**To the Editor:** A small outbreak of primary pneumonic plague took place in the Shimla District of Himachal Pradesh State in northern India during February 2002. Sixteen cases of plague were reported with a case-fatality rate of 25% (4/16). The infection was confirmed to the molecular level with PCR and gene sequencing (*1*). A previous outbreak in this region during 1983 was suggestive of pneumonic plague (22 cases, 17 deaths) but was not confirmed. In India, the last laboratory-confirmed case of plague was reported in 1966 from Karnataka State (*2*).

The index patient for the 2002 outbreak lived in a hamlet in the Himalayas. He went hunting on January 28, 2002, in a nearby forest at a height of ≈500–600 m from his house. There, he killed a sick wild cat and skinned it. He returned home on February 2 and sought treatment for fever, chills, and headache. On February 4, breathlessness, chest pain, and hemoptysis developed; radiologic findings were suggestive of lobar pneumonia, and treatment with augmentin was begun. He died the next day. Subsequently, 13 of his relatives exhibited a similar illness, although 2 additional patients acquired infection in the hospital. The incubation period for those patients was 1–4 days, which is consistent with that of pneumonic plague. 

A team of microbiologists, epidemiologists, and entomologists visited the village after 7 more cases were reported until February 12, 2002, followed by a team from the National Institute of Communicable Diseases (NICD), New Delhi. The following case-patient definition was used: a person who sought treatment for fever of rapid onset, chills, chest pain, breathlessness, headache, prostration, and hemoptysis. A total of 16 cases were reported from 3 hospitals in the area: a local civil hospital, the state medical college, and a regional tertiary care hospital. Clinical material collected from the case-patients and their contacts was initially processed in the laboratories of these hospitals. Wayson staining provided immediate presumptive diagnosis, and confirmatory tests were performed at NICD. Diagnosis of plague was confirmed for 10 (63%) of 16 patients (*1*).

NICD conducted the following laboratory tests on 2 suspected culture isolates, 2 sputum specimens, 1 lung autopsy material specimen, and 1 lung lavage sample (Table): 1) direct fluorescent antibody test for *Yersinia pestis*; 2) culture and bacteriophage lysis test; and 3) PCR and gene sequencing to detect *Y. pestis*–specific genes (*pla* and *F1*). All these tests confirmed that isolates were *Y. pestis* and met all the World Health Organization’s recommended criteria (*3*). 

**Table:** Epidemiologic characteristics and laboratory findings of patients with suspected cases of pneumonic plague, India, 2002 (*1*)*

Patient no.	Relation to index patient	Age, y/sex	Onset of symptoms	Outcome	Wayson staining	Blood c/s	Sputum c/s	Molecular results	Serologic results
1	Index patient	35/M	Feb 2	Died Feb 5	–	–	–	–	–
2	Wife	29/F	Feb 6	Died Feb 14	–	–	–	Confirmed	Single sample positive
3	Brother	26/M	Feb 7	Discharged Mar 8	–	*Yersinia pestis*	–	Confirmed	Negative
4	Sister	31/F	Feb 9	Died Feb 18	–	*Y. pestis*	–	Confirmed	–
5	Sister	27/F	Feb 12	Discharged Feb 25	–	–	–	–	Negative
6	Brother-in-law	35/M	Feb 12	Discharged Mar 8	–	–	–	–	Negative
7	Brother-in-law	35/M	Feb 10	Discharged Feb 21	–	–	–	–	Negative
8	Sister-in-law	38/F	Feb 9	Discharged Feb 25	–	–	–	–	>4-fold rise
9	Companion on hunting trip	36/M	Feb 10	Discharged Feb 28	–	–	–	–	Same titer in paired serum specimens
10	Sister-in-law	37/F	Feb 12	Discharged Mar 11	–	–	–	–	>4-fold rise
11	Relative of sister-in-law	40/F	Feb 12	Died Feb 14	Positive	*Y. pestis*	*Y. pestis*	Confirmed	Negative
12	Aunt	57/F	Feb 10	Discharged Mar 4	Positive	Negative	*Y. pestis*	Negative	>4-fold rise
13	Neighbor	46/F	Feb 11	Discharged Feb 27	–	–	–	–	>4-fold rise
14	Son of neighbor	22/M	Feb 8	Discharged Feb 27	–	–	–	–	>4-fold fall
15	Patient hospitalized with epilepsy	47/F	Feb 11	Discharged Feb 18	–	–	–	–	Negative
16	Husband/atten- dant of patient 15	60/M	Feb 11	Discharged Mar 11	Positive	*Y. pestis*	*Y. pestis*	–	>4-fold rise

*c/s, culture/sensitivity; –, sample not submitted; paired serum samples were tested 4 weeks apart.

Antibodies against F1 antigen of *Y. pestis* were detected by passive hemagglutination testing of paired serum samples. Although 5 patients showed a >4-fold rise, 1 patient showed a >4-fold fall in antibody titer. In contrast, samples from 6 patients were negative for *Y. pestis*, and no change was found in the titers from 1 patient. No serum sample was collected from the index patient; for the 2 other patients who died, 1 of the single serum samples became contaminated, and the other was positive for *Y. pestis* (*4*). Paired serum samples from the case-patients were collected on a single day 4 weeks apart during the visit of the NICD team, regardless of the duration of symptoms.

Antimicrobial drug sensitivity testing was carried out by the Kirby-Bauer disk diffusion method. All isolates were sensitive to doxycycline, tetracycline, chloramphenicol, streptomycin, ciprofloxacin, gentamicin, and amikacin but were resistant to penicillin.

No fleas or other ectoparasites were found on the 6 cats, 8 dogs, 6 cows, 4 calves or 2 trapped rodents in the village. One serum sample, with pooled blood from 3 dogs was negative for antibodies against F1 antigen. Before these infections occurred, a heavy snowfall in the region had reduced the activity of rodents and was unfavorable for the survival and multiplication of rat fleas. The snow also helped restrict the spread of the infection because of reduced movement of the local population (*1*).

Primary pneumonic plague is acquired by inhaling infective droplets from persons or animals and rarely by accidental aerosol exposure. *Y. pestis* is a category A agent of bioterrorism (*5*). It is not truly airborne; person-to-person transmission requires face-to-face exposure within 2 m of a coughing patient (*2*). During 1977–1998, in the western United States, 23 cases of cat-associated human plague were reported. Bites, scratches, or other contact with infectious material while handling infected cats resulted in 17 cases of bubonic plague, 1 case of primary septicemic plague, and 5 cases of primary pneumonic plague (*6*).

In our report, close and prolonged contact with the index patient while providing care (for example, wiping his face during hemoptysis, supporting him during a bout of coughing, taking him to the hospital in a vehicle) resulted in secondary cases. Because of the severe winter, poor ventilation in houses further helped the illness spread. All patients acquired infection before plague was suspected. Initially, patients were treated for community-acquired pneumonia, which delayed the proper treatment and led to deaths. A patient admitted for status epilepticus was infected by her attendant, who in turn, acquired infection from a terminally ill plague patient for whom he provided some care. The patient with epilepsy and her attendant shared a common room with the terminally ill wife of the index patient, which was small and poorly ventilated. Surprisingly, the relative of the index case-patient who had accompanied him to the forest survived the infection; whereas, the wife and sister of the index patient died. No spread to healthcare workers was noted.

When plague was suspected immediate preventive measures were taken, for example, fumigation of the index patient’s residence and any vehicles used for transporting the patients; active surveillance and education; standard work precautions; chemoprophylaxis for patient contacts and paramedics; and isolation and treatment of patients (*1*). The transmission rate for primary pneumonic plague is relatively low compared with that of many other communicable diseases; the average number of secondary cases per primary case is 1.3, according to a study done by Gani and Leach (*7*).

The key element in the control of small outbreaks of primary pneumonic plague could be the intensity of disease surveillance system (*7*). As a result, the state government has established a Plague Surveillance Unit in District Shimla of Himachal Pradesh in 2002 (*1*).
